# Construction of a telomere-related gene signature to predict prognosis and immune landscape for glioma

**DOI:** 10.3389/fendo.2023.1145722

**Published:** 2023-06-02

**Authors:** Qin Xie, Tingting Liu, Xiaole Zhang, Yanli Ding, Xiaoyan Fan

**Affiliations:** ^1^ Department of Neurosurgery, Hangzhou Ninth People’s Hospital, Hangzhou, Zhejiang, China; ^2^ Department of Endocrinology, Affiliated Haikou Hospital of Xiangya School of Central South University, Haikou, Hainan, China; ^3^ Intensive Care Unit, Hangzhou Ninth People’s Hospital, Hangzhou, Zhejiang, China

**Keywords:** glioma, signature, immunotherapy, checkpoint, clinical treatment

## Abstract

**Background:**

Glioma is one of the commonest malignant tumors of the brain. However, glioma present with a poor clinical prognosis. Therefore, specific detection markers and therapeutic targets need to be explored as a way to promote the survival rate of BC patients. Therefore, we need to search for quality immune checkpoints to support the efficacy of immunotherapy for glioma.

**Methods:**

We first recognized differentially expressed telomere-related genes (TRGs) and accordingly developed a risk model by univariate and multivariate Cox analysis. The accuracy of the model is then verified. We evaluated the variations in immune function and looked at the expression levels of immune checkpoint genes. Finally, to assess the anti-tumor medications often used in the clinical treatment of glioma, we computed the half inhibitory concentration of pharmaceuticals.

**Results:**

We finally identified nine TRGs and built a risk model. Through the validation of the model, we found good agreement between the predicted and observed values. Then, we found 633 differentially expressed genes between various risk groups to identify the various molecular pathways between different groups. The enrichment of CD4+ T cells, CD8+ T cells, fibroblasts, endothelial cells, macrophages M0, M1, and M2, mast cells, myeloid dendritic cells, and neutrophils was favorably correlated with the risk score, but the enrichment of B cells and NK cells was negatively correlated with the risk score. The expression of several immune checkpoint-related genes differed significantly across the risk groups. Finally, in order to create individualized treatment plans for diverse individuals, we searched for numerous chemotherapeutic medications for patients in various groups.

**Conclusion:**

The findings of this research provide evidence that TRGs may predict a patient’s prognosis for glioma, assist in identifying efficient targets for glioma immunotherapy, and provide a foundation for an efficient, customized approach to treating glioma patients.

## 1 Introduction

According to the World Health Organization, glioma is one of the most prevalent malignant tumors of the brain and is categorized as grades 1, 2, 3, or 4; grades 1 and 2 are low grade glioma (LGG), while grades 3 and 4 are high grade glioma (glioblastoma multiforme, GBM) (1, 2). 30% of all primary brain and spinal cord tumors are glioma, which make up more than 80% of all malignant brain tumors and are clinically very likely to be fatal (3). Glioma currently have a poor clinical prognosis upon presentation. Despite advancements in chemotherapeutic agents, radiation, and surgical methods for resecting tumors, the overall survival of glioma patients is still not encouraging (4). After conventional surgery, radiation and chemotherapy, glioma patients have a median survival period of about 14 months and an estimated 5-year survival rate of about 9.8% (5). So far, immunotherapy for glioma is the more effective treatment modality. Immune checkpoint inhibitor therapy allows effector T cells to reactivate and exert cytotoxicity on tumor cells through a combination of specific antibodies and checkpoint molecules (10.3389/fimmu.2020.578877). Therefore, we need to search for quality immune checkpoints to support the efficacy of immunotherapy for glioma.

Telomere is a region at the end of a chromosome that is composed of two parts, the repetitive TTAGGG DNA sequence and the shieldin complex (6). Telomeres ensure the stability of chromosomes, providing security, and are significant for cell division and certain diseases (7). In addition, telomere abnormalities can lead to many diseases and are closely associated with the development of many mental health problems and cancer (7, 8). A study elucidated polymorphisms in telomere length-related genes and found that some telomeric loci were associated with a high risk of liver cancer (9). It has been shown that the length of the telomere-related genes (TRGs) is associated with the development of glioma (10). Malignant glioma usually exhibit telomerase activity, although normal brain tissue hardly ever does (11). Malignant glioma cells may be capable of unrestricted proliferation and apoptosis inhibition due to abnormal telomerase reactivation (11).

In the research, we screened and correlated TRGs with the aim of identifying immune checkpoints associated with glioma immunotherapy to improve the efficacy of clinical glioma and improve patient survival.

## 2 Materials and methods

### 2.1 Preparation of data

The TCGA-glioma and GEO-GSE74187 databases provided the RNA-seq data and clinical information for glioma (12). Data that was missing or had a survival time of less than 30 days was removed. TRGs were downloaded from TelNet (http://www.cancertelsys.org/telnet/; **Table S1**) (13).

### 2.2 Construction and validation of model

To find TRGs that were differently expressed between normal and glioma samples (|logFC| >= 1 and *P* value< 0.05), the R package limma and wilcox tests were used (14). Prognostic TRGs were identified using univariate Cox analysis (*P<* 0.001), and a risk model was created using multivariate Cox analysis. Each patient with glioma had their risk score calculated using a formula: $\sum_{i=1}^{k}\beta iSi$ . To validate this model, the GEO-GSE74187 dataset was used as an external validation set. To compare the survival rates of various groups, a Kaplan-Meier analysis was used. To evaluate the accuracy of survival prediction, the receiver operating characteristic (ROC) curves and the area under curve (AUC) were used.

Based on clinical characteristics, we divided the patients into several groups and investigated the survival rates of various groups within various groupings. The model was tested using univariate and multivariate Cox analyses to ensure that it was an accurate predictor of prognosis. The consistency index (C-index) was used to calculate the model’s accuracy. A nomogram was developed to predict the 1, 3, and 5-year survival rates of glioma patients using the model and clinical data. We found differentially expressed genes (DEGs) in different groups (|logFC > 1| and FDR< 0.05) and ran kyoto encyclopedia of genes and genomes (KEGG) and Gene Ontology (GO) enrichment analyses on these DEGs (*P<* 0.05) using clusterProfiler 4.0 (15).

### 2.3 Evaluation of immune landscape

The number of gene mutations was determined using mutational analysis, and scores for tumor immune dysfunction and exclusion (TIDE) and tumor mutation burden (TMB) were computed to forecast immunotherapy response (16, 17). Additionally, we computed survival variations between various TMB groups and other groups. Immune cell infiltration was calculated using the EPIC, TIMER, MCP-COUNTER, XCELL, QUANTISEQ, CIBERSORT, and CIBERSORT-ABS algorithms (18–24). To evaluate the variations in immune function and look into the expression levels of several immunological checkpoint genes, we used a single-sample gene set enrichment analysis (ssGSEA).

### 2.4 Identification of anti-tumor drugs

To assess the anti-tumor medications often used in the clinical treatment of glioma, we calculated the half inhibitory concentration (IC50) of pharmaceuticals and compared the IC50 across various groups using the “pRRophetic” R package (10.1371/journal.pone.0107468).

## 3 Result

### 3.1 Construction and validation of signature

**Figure 1:**
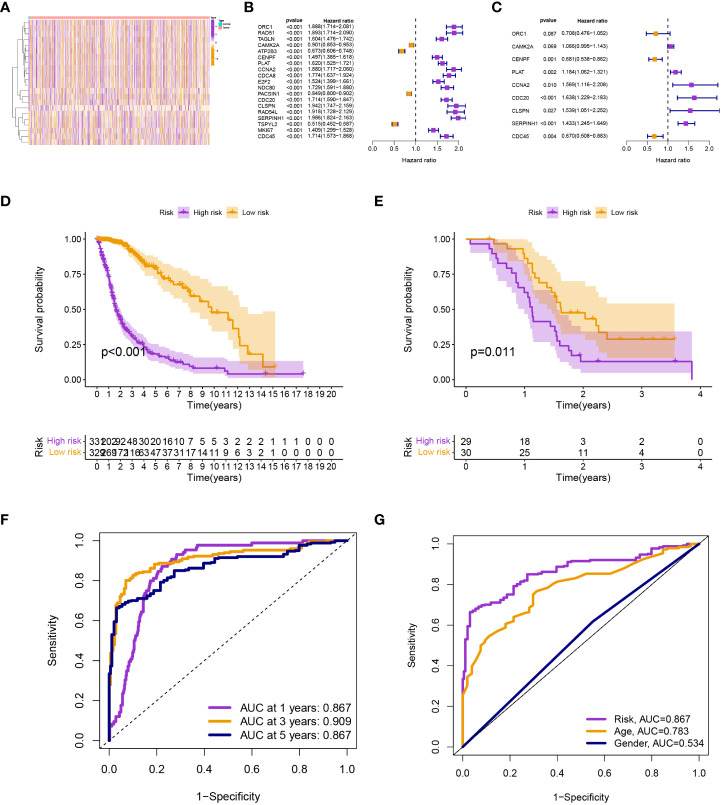
**(A)** Differential expression analysis. **(B)** and **(C)** Univariate and multivariate Cox analyses. **(D)** and **(E)** The survival analysis from TCGA-glioma and GSE74187. **(F)** The AUC values for the model. **(G)** The AUC of the model was also higher than other clinical features.

Differential expression analysis revealed 22 differentially expressed TRGs (**Figure 1A**), univariate Cox analysis revealed 19 prognostic TRGs (*P<* 0.001; **Figure 1B**), and multivariate Cox analysis produced a signature with 9 prognostic TRGs (**Figure 1C**). The findings of the survival analysis (*P<* 0.001; **Figure 1D**) and the validation set from GSE74187 (*P* = 0.011; **Figure 1E**) both indicated that the high-risk group had a shorter survival time. The signature was used to forecast glioma patients’ 1-, 3-, and 5-year survival rates, with the corresponding AUC values of 0.867, 0.909, and 0.867 (**Figure 1F**). Compared to other clinical features, the model’s AUC was greater, indicating that it is more trustworthy (**Figure 1G**).

**Figure 2:**
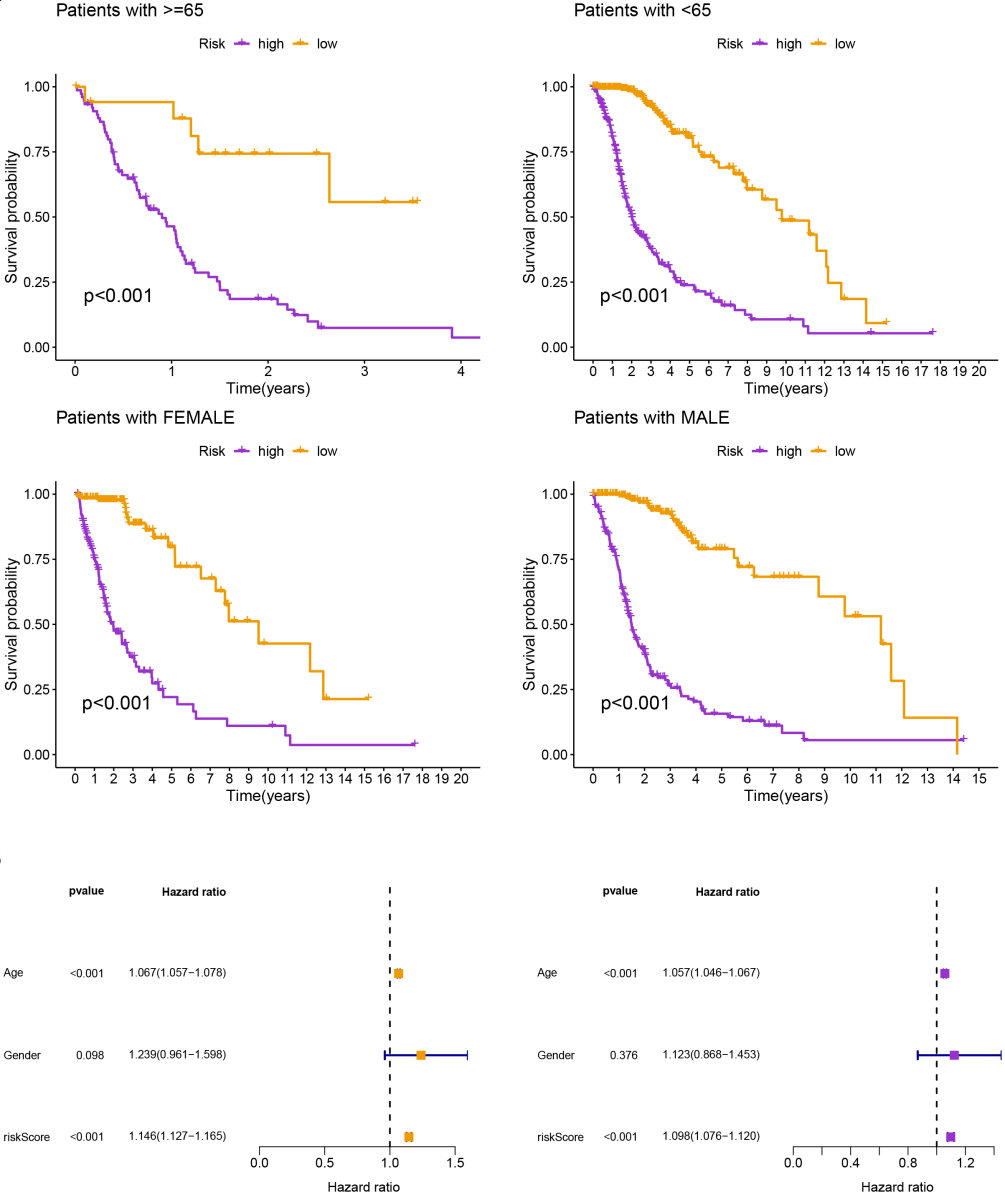
**(A)** According to the various clinical subgroups, patients in the low-risk group had a longer survival time. **(B)** It was discovered that the risk score was a standalone prognostic factor.

**Figure 3:**
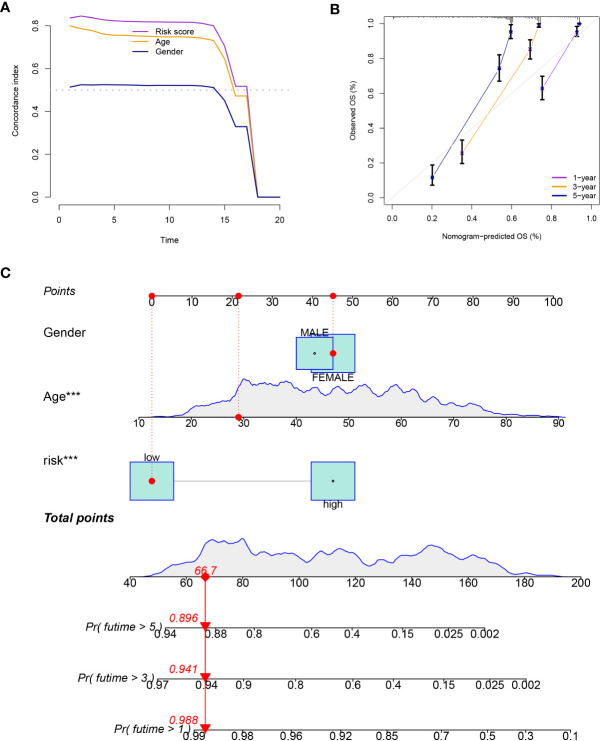
**(A)** The model performed better in predicting the prognosis of glioma than did traditional clinical criteria. **(B)** The observed survival rates demonstrated a strong agreement with the projected rates in the correlation plot. **(C)** A nomogram with signature and clinical characteristics.

Patients in the low-risk group had a longer survival time, according to the various clinical subgroups, suggesting that the model is applicable to patients with a range of clinical features (**Figure 2A**). In both univariate and multivariate Cox analyses, the risk score was shown to be an independent prognostic predictor (*P<* 0.001; **Figures 2B**). The C-index showed that the model performed better in predicting the prognosis of glioma than did traditional clinical criteria (**Figure 3A**). The correlation plot showed that the observed 1, 3, and 5-year survival rates and the anticipated rates agreed strongly (**Figure 3B**). We developed a nomogram using the signature and clinical characteristics that might be used to precisely forecast the survival of glioma patients (**Figure 3C**).

### 3.2 Assessment of immunological landscape

**Figure 4:**
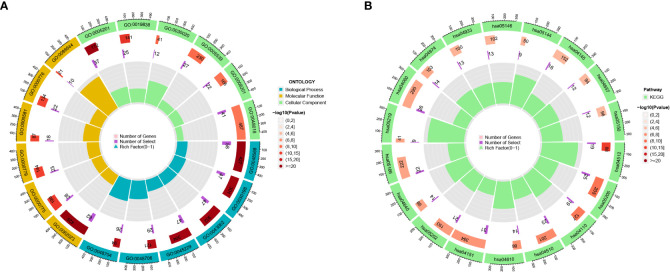
**(A, B)** The GO and KEGG analyses for 633 DEGs.

**Figure 5:**
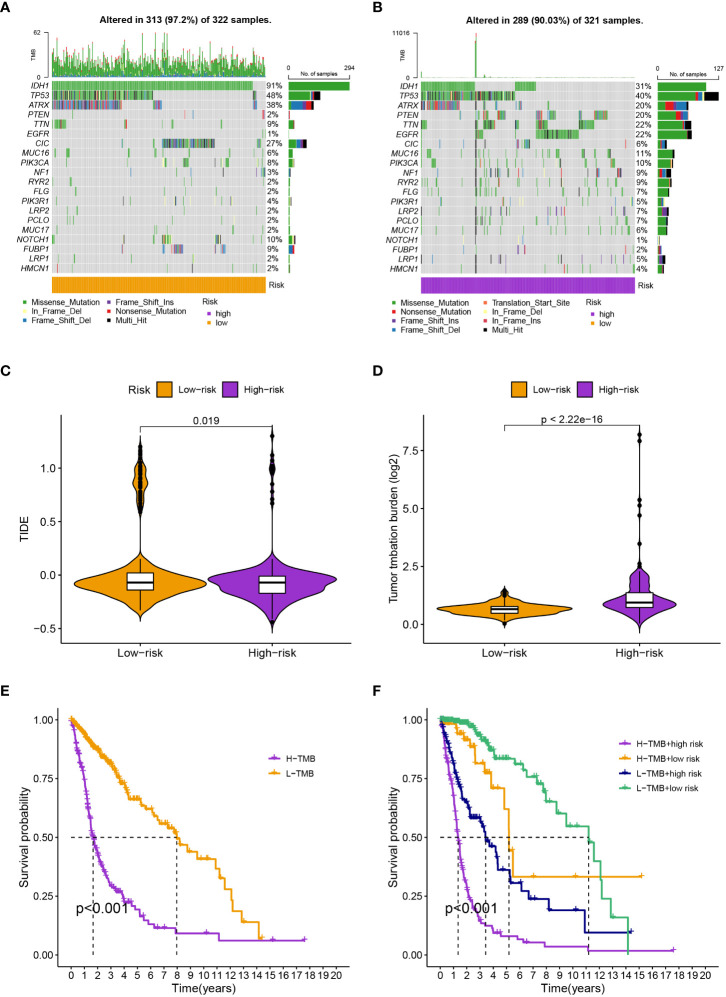
**(A, B)** The specific genes that have been altered differed substantially between groups. **(C)** and **(D)** The high-risk group have a lower TIDE score and a higher TMB score. **(E)** High-TMB groups had considerably reduced survival rates. **(F)** The four groups’ survival rates varied greatly from one another.

We found 633 DEGs between various risk groups to analyze the various molecular pathways between different groups (**Table S2**). **Figures 4A, B** show the results of the GO and KEGG analyses, while **Tables S3**, **S4** give more information. In comparison to the high-risk group, the frequency of gene mutations was much greater in the low-risk group (**Figures 5A, B**). Lower TIDE scores (*P* = 0.019; **Figure 5C**) and higher TMB scores (*P<* 0.001; **Figure 5D**) in the high-risk group indicated that they were more likely to respond to immunotherapy. According to survival research, distinct TMB and risk groups had statistically different survival rates, suggesting that integrating TMB scores might improve the ability to predict the prognosis of glioma patients (**Figures 5E, F**).

**Figure 6:**
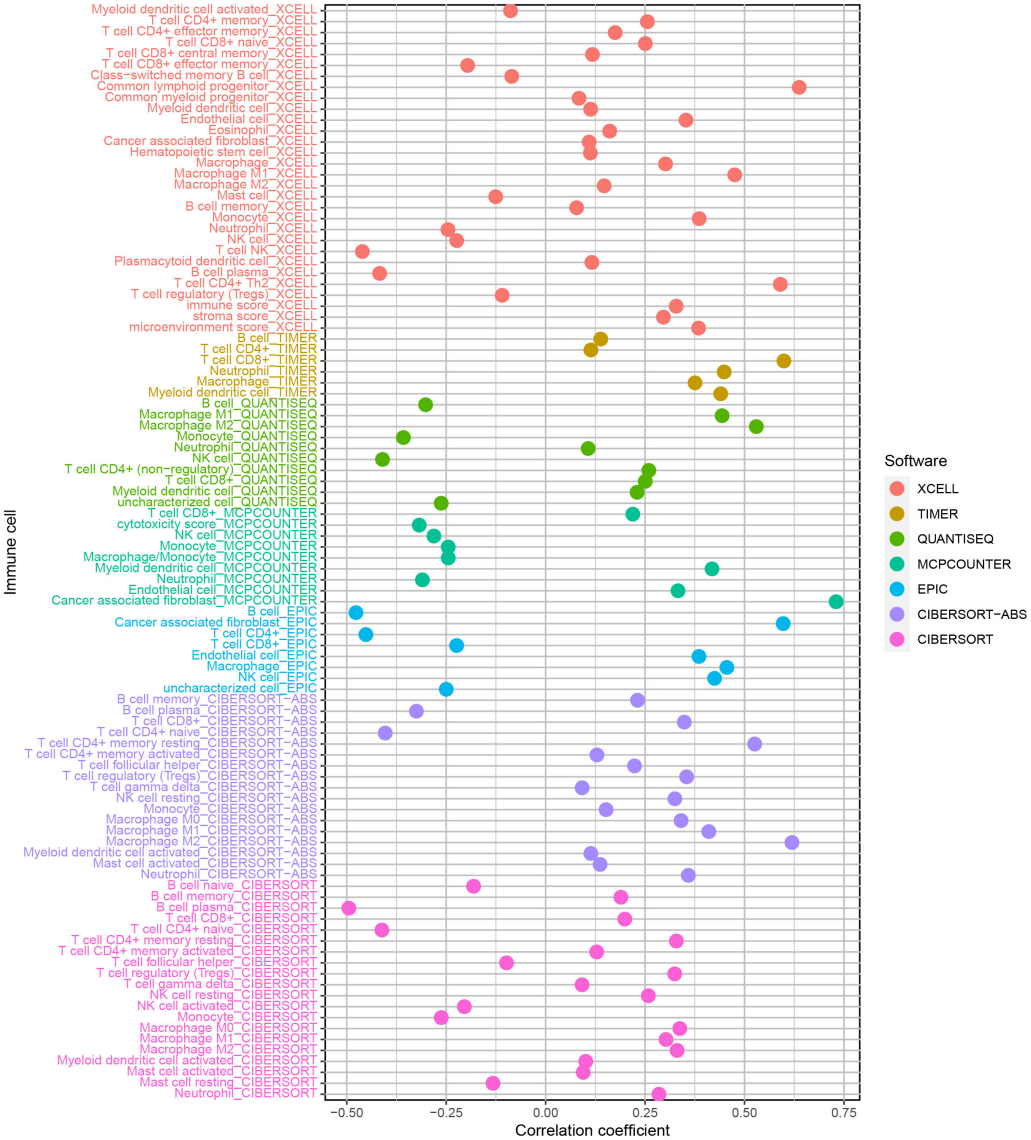
The enrichment of CD4+ T cells, CD8+ T cells, fibroblasts, endothelial cells, macrophages M0, M1 and, M2, mast cells, myeloid dendritic cells, and neutrophils was favorably correlated with the risk score, but the enrichment of B cells and NK cells was negatively related to the risk score.

**Figure 7:**
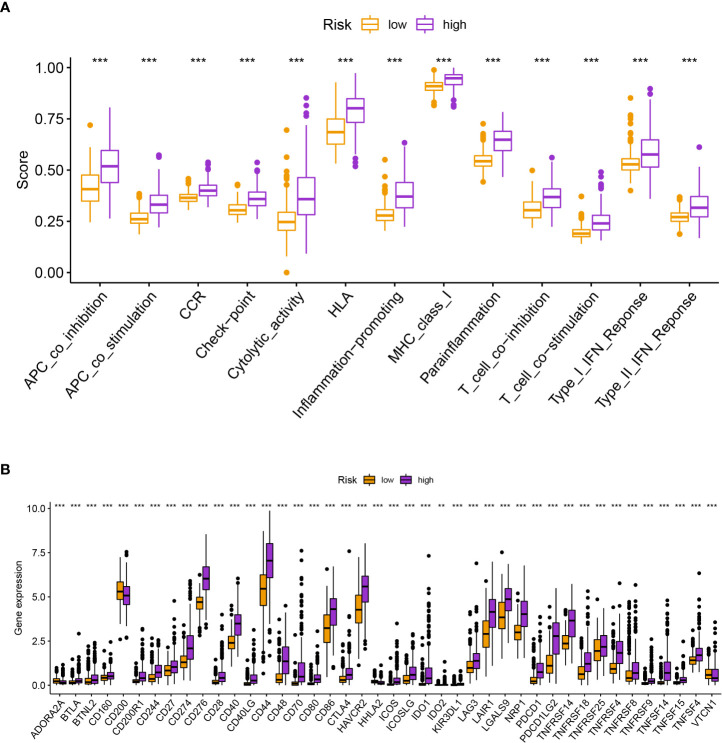
**(A)** The various risk groups showed statistically significant differences in all immunological activities. **(B)** The expression of immune checkpoint-related genes differed significantly across the risk groups. *** means P < 0.001.

The enrichment of CD4+ T cells, CD8+ T cells, fibroblasts, endothelial cells, macrophages M0, M1, and, M2, mast cells, myeloid dendritic cells, and neutrophils was favorably correlated with the risk score, but the enrichment of B cells and NK cells was negatively correlated with the risk score (**Figure 6**). The various risk groups showed statistically significant differences in all immunological activities (**Figure 7A**). The expression of several immune checkpoint-related genes, such as CTLA-4 (*P<* 0.001), PDCD1 (*P<* 0.001), LAG3 (*P<* 0.001), and CD274 (*P<* 0.001), differed significantly across the risk groups (**Figure 7B**).

### 3.3 Selection of anti-tumor drugs

**Figure 8:**
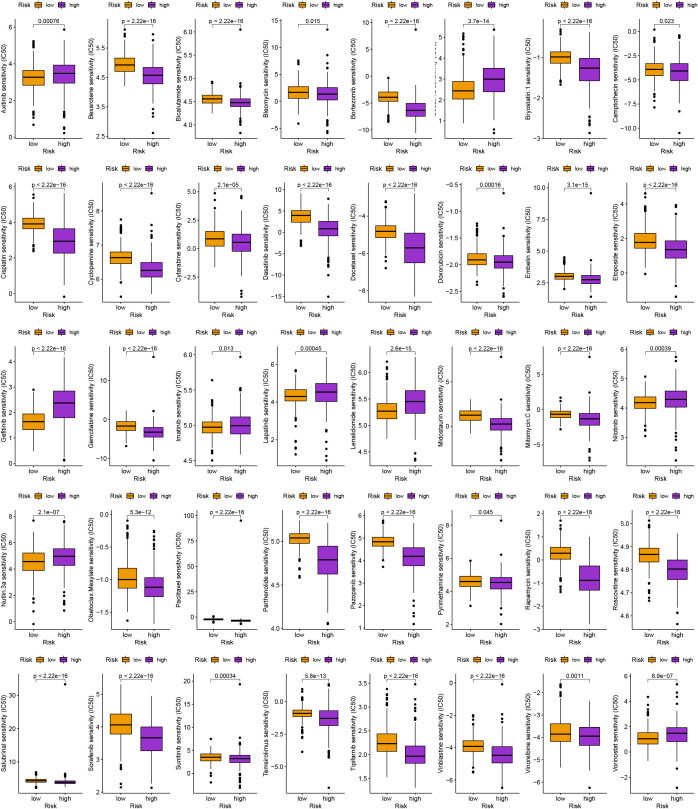
Identification of traditional chemotherapy medications.

Along with immunotherapy, we are looking for chemotherapeutic drugs for patients in different risk groups. Finally, in order to create individualized treatment plans for diverse individuals, we searched for numerous chemotherapeutic medications for patients in various groups (*P<* 0.001; **Figure 8**).

## 4 Discussion

With a low patient survival rate and a poor clinical prognosis, glioma has a high occurrence (25). The overall survival rate of glioma patients continues to be dismal despite the quick development of surgical resection methods, chemotherapy, and radiation (4). Therefore, to ensure that immunotherapy for glioblastoma is effective, we must discover superior immune checkpoints.

High-throughput sequencing data and computational biology are currently used extensively in the study of biomedicine (26, 27). Wang et al., for instance, identified biomarkers in several tumors using computational biology techniques like WGCNA, which gives us a methodologically sound foundation on which to examine the process of carcinogenesis (28–30). In the research, we first created a risk model linked to TRGs by discovering TRGs. After that, using this methodology to determine the risk score for glioma patients, we divided them into low- and high-risk groups. In order to confirm the validity of the model, we did univariate and multivariate Cox analyses on the patients in the high-risk group and discovered that they all had shorter survival rates than those in the low-risk group. As expected, the predictive accuracy of the risk model was high.

Then, we analyzed the immune infiltration in the high-risk and low-risk groups and found that the enrichment of CD4+ T cells, CD8+ T cells, fibroblasts, endothelial cells, macrophages M0, M1 and, M2, mast cells, myeloid dendritic cells, and neutrophils was favorably correlated with the risk score, but the enrichment of B cells and NK cells was negatively related to the risk score. CD8+ T cells are a common type of T cells, and the CD8+ T cell family establishes a neuronal-immune-cancer axis through midkine activation to enhance favorable conditions for the growth of low-grade glioma (31). In addition, in one study performed by Ge. et al, related discussions elucidated that macrophages, neutrophils and fibroblasts can be regulated by TP53I13, altering tumor immune infiltration and thus promoting glioma development and metastasis (32). The potential of neutrophils as therapeutic targets in cancer biology has now been extensively studied. Neutrophils play a complex role in cancer, including their ability to exert pro- or anti-tumor activity (33). However, further studies are needed to investigate their exact roles and mechanisms of action to develop targeted therapeutic approaches. Furthermore, although the degree of neutrophil infiltration correlates with glioma grade, the underlying mechanisms are unknown (33).

In addition, in our study, we also found some significant TRGs such as CTLA-4, PDCD1, LAG3, and CD274 (PD-L1). A critical part of the tumor immune response pathway is played by CTLA-4 (34). Although it has been shown that CTLA-4 positively correlates with immune-related proteins in glioma, excessive CTLA-4 expression is associated with a worse prognosis for glioma patients (35). An immunoglobulin superfamily cell surface membrane protein, encoded by the PDCD1 gene, is responsible for programmed cell death. Activated monocytes, NK cells, T cells, and B cells are the main cell types that express it. Additionally, B or T cell receptor signaling can cause PDCD1 expression, and tumor necrosis factor stimulation can further increase it (36). LAG3, an inhibitory receptor that is predominantly located on activated immune cells and is frequently co-expressed with PD-1 on depleted T cells, has emerged as a crucial immunomodulator target (37). CD274 (PD-L1) is considered a major prognostic biomarker for immunotherapy of many cancers. CD274 (PD-L1) is not only associated with decreased cytotoxic T lymphocytes and increased Tregs in glioma lesions, but also has an intrinsic oncogenic effect through interaction with Ras (10.3389/fphar.2018.01503). It has been shown that LAG3 is realized to be highly expressed in glioma patients, but the sample size is small and further experimental validation is needed (38). In addition, in addition to immunotherapy, we have studied a large number of drugs for different groups of patients in order to develop individualized treatment plans.

Although we tried to avoid errors as much as possible to make our experiments credible, there are still some shortcomings that need to be improved. First, due to database limitations, we were unable to accurately compare the corresponding checkpoint inhibitor IC50. In addition, we did not conduct simultaneous *in vitro* experimental validation, and further in-depth experiments are needed for this part. We believe that our risk model construction is reasonable and acceptable for further validation in future clinical trials based on the above analysis, validation, and previous relevant reports. Most importantly, the current data is limited and we need to collect more data from the clinic to expand the database for future studies.

## Conclusion

The present study support that TRGs could predict the prognosis of glioma patients and help to find effective targets for glioma immunotherapy and can serve as a basis for effective individualized treatment of glioma patients.

## Data availability statement

The datasets presented in this study can be found in online repositories. The names of the repository/repositories and accession number(s) can be found in the article/**Supplementary Material**.

## Ethics statement

This study was reviewed and approved by the Ethics Committee of Hangzhou Ninth People's Hospital.

## Author contributions

QX and TL designed the study. QX, TL, XZ, YD, and XF performed data analysis. QX and TL drafted and revised the manuscript. All authors read and approved the final manuscript.

## Supplementary material

Supplementary Table 1 The TRGs.

Supplementary Table 2 The 33 DEGs.

Supplementary Table 3 The GO enrichment analysis.

Supplementary Table 4 The KEGG enrichment analysis.
